# Supplementary material for: Causal Relationship between Aging and Anorexia Nervosa: A White-Matter-Microstructure-Mediated Mendelian Randomization Analysis
Source: Biomedicines. 2024 Aug 16;12(8):1874. doi: 10.3390/biomedicines12081874 (PMC11351342; doi:10.3390/biomedicines12081874)
Supplement: Supplementary file 1 [file biomedicines-12-01874-s001.zip › Supplementary Figure/Figure S4/Singe SNP plot of MR analysis for TL to ubm-b-1672.pdf]

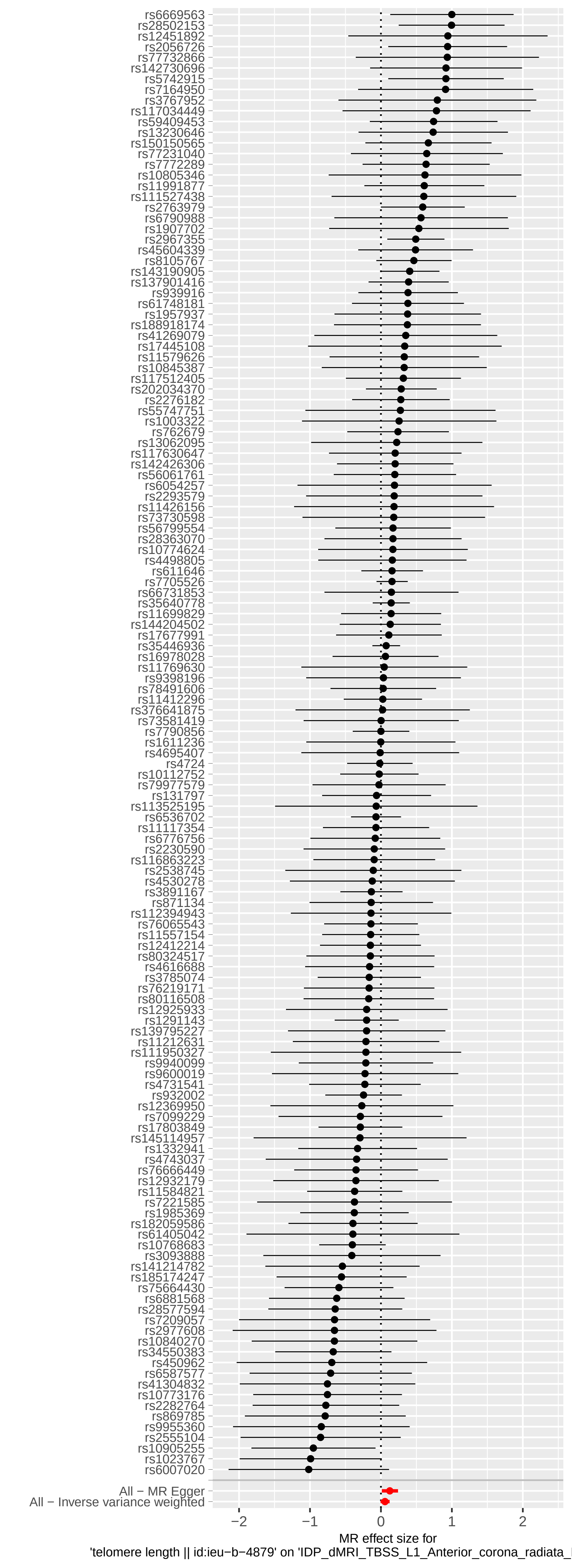
rs6669563
rs28502153
rs12451892
rs2056726
rs77732866
rs142730696
rs5742915
rs7164950
rs3767952
rs117034449
rs59409453
rs13230646
rs150150565
rs77231040
rs7772289
rs10805346
rs11991877
rs111527438
rs2763979
rs6790988
rs1907702
rs2967355
rs45604339
rs8105767
rs143190905
rs137901416
rs939916
rs61748181
rs1957937
rs188918174
rs41269079
rs17445108
rs11579626
rs10845387
rs117512405
rs202034370
rs2276182
rs55747751
rs1003322
rs762679
rs13062095
rs117630647
rs142426306
rs56061761
rs6054257
rs2293579
rs11426156
rs73730598
rs56799554
rs28363070
rs10774624
rs4498805
rs611646
rs7705526
rs66731853
rs35640778
rs11699829
rs144204502
rs17677991
rs35446936
rs16978028
rs11769630
rs9398196
rs78491606
rs11412296
rs376641875
rs73581419
rs7790856
rs1611236
rs4695407
rs4724
rs10112752
rs79977579
rs131797
rs113525195
rs6536702
rs11117354
rs6776756
rs2230590
rs116863223
rs2538745
rs4530278
rs3891167
rs871134
rs112394943
rs76065543
rs11557154
rs12412214
rs80324517
rs4616688
rs3785074
rs76219171
rs80116508
rs12925933
rs1291143
rs139795227
rs11212631
rs111950327
rs9940099
rs9600019
rs4731541
rs932002
rs12369950
rs7099229
rs17803849
rs145114957
rs1332941
rs4743037
rs76666449
rs12932179
rs11584821
rs7221585
rs1985369
rs182059586
rs61405042
rs10768683
rs3093888
rs141214782
rs185174247
rs75664430
rs6881568
rs28577594
rs7209057
rs2977608
rs10840270
rs34550383
rs450962
rs6587577
rs41304832
rs10773176
rs2282764
rs869785
rs9955360
rs2555104
rs10905255
rs1023767
rs6007020
All − MR Egger
All − Inverse variance weighted
−2
−1
0
1
2
MR effect size for
'telomere length || id:ieu−b−4879' on 'IDP_dMRI_TBSS_L1_Anterior_corona_radiata_
